# Clinical features and power spectral entropy of electroencephalography in Wilson's disease with dystonia

**DOI:** 10.1002/brb3.2791

**Published:** 2022-10-25

**Authors:** Shaoru Zhang, Aiqun Liu, Zhihua Zhou, Zheng Huang, Jing Cheng, Danping Chen, Qizhi Zhong, Qingyun Yu, Zhongxing Peng, Mingfan Hong

**Affiliations:** ^1^ Department of Neurology The First Affiliated Hospital, Clinical Medicine College of Guangdong Pharmaceutical University Guangzhou Guangdong China

**Keywords:** dystonia, electroencephalography, executive dysfunction, power spectral entropy (PSE), Wilson's disease

## Abstract

**Objective:**

To study the clinical features and power spectral entropy (PSE) of electroencephalography signals in Wilson's disease (WD) patients with dystonia.

**Methods:**

Several scale evaluations were performed to assess the clinical features of WD patients. Demographic information and electroencephalography signals were obtained in all subjects.

**Results:**

34 WD patients with dystonia were recruited in the case group and 24 patients without dystonia were recruited in the control group. 20 healthy individuals were included in the healthy control group. The mean body mass index (BMI) in the case group was significantly lower than that in the controls (*p* < .05). The case group had significantly higher SAS, SDS, and Bucco‐Facial‐Apraxia Assessment scores (*p* < .05). Total BADS scores in the case group were lower than those in the control group (*p* < .01). Note that 94.11% of the case group presented with dysarthria and 70.59% of them suffered from dysphagia. Dysphagia was mainly related to the oral preparatory stage and oral stage. Mean power spectral entropy (PSE) values in the case group were significantly different (*p* < .05) from those in the control group and the healthy group across the different tasks.

**Conclusions:**

The patients with dystonia were usually accompanied with low BMI, anxiety, depression, apraxia, executive dysfunction, dysarthria and dysphagia. The cortical activities of the WD patients with dystonia seemed to be more chaotic during the eyes‐closed and reading tasks but lower during the swallowing stages than those in the control group.

## Graphical abstract

WD patients with dystonia were more susceptible to suffer from low BMI and highߚlevel neurological dysfunctions. It was the first time to apply the PSE of EEG to detect the cortical synaptic activity in WD patients. The cortical activities of the WD patients with dystonia seemed to be more chaotic during the eyes‐closed and reading tasks but lower during the swallowing stages than those in the control group.

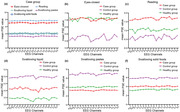

## 1 INTRODUCTION

Wilson's disease (WD) is an autosomal recessive disease related to *ATP7B* mutations causing copper transport disorder (Sandahl et al., 2021). Disease features correlate with the organs or tissues affected, which are mainly the liver and brain leading to hepatic cirrhosis, dysarthria, and dysphagia (Dusek et al., 2019). In addition, WD patients suffer from anxiety, depression, or other neurological symptoms (Leśniak et al., 2022). The percentage of WD patients with dystonia has been reported to range from 11% to 65% (Członkowska et al., 2018). Clinically, the manifestations of WD patients with dystonia seem to be more severe. Yet, there have been few systematic evaluations of WD patients with dystonia.

The view that dystonia is a neural network dysfunction involving the basal ganglia‐cerebello‐thalamo‐cortical circuit have emerged as standouts in recent years (Balint et al., 2018; Ganguly et al., 2021). However, little is known about the neural activities of WD patients with dystonia. Electroencephalography (EEG) recording is a noninvasive method with high temporal resolution to quantify electrical neural activities (Li et al., 2020; Zhang et al., 2021). Since neural activity is irregular and nonperiodic, the application of nonlinear dynamics is developed to quantitatively analyze these complex and dynamic signals (Hou et al., 2021). As an effective application of nonlinear dynamics, power spectral entropy (PSE) is estimated on the basis of power spectral density to quantitative analyses of individuals’ cortical activities during different tasks both in the temporal domain and frequency domain (Deutschová et al., 2021; Tian et al., 2017). Thus, PSE may take advantage to evaluating the characteristics of the neural activities among the WD patients with dystonia.

In general, the neurobehavioral disabilities and cortical activities in WD patients with dystonia are poorly understood and further research is extremely meaningful. We undertook this study to clarify the clinical features and applied PSE of EEG to describe the neural activities of WD patients with dystonia.

## 2 METHODS

This single‐center, prospective, and observational comparative study was conducted from October 8, 2019 to January 31, 2021 and approved by the Ethics Committee of the First Affiliated Hospital of Guangdong Pharmaceutical University. All participants were informed of the aims of the study and signed informed consent forms.

### 2.1 Participants

The patients met the diagnostic criteria for WD approved by the American Association for the Study of Liver Diseases (Roberts & Schilsky, 2008). Patients were excluded if they had evidence of coexisting epilepsy, hyperammonemia or schizophrenia. Further exclusion criteria were as follows: (1) other structural brain abnormalities, such as focal infarction, microhemorrhages, vascular malformations, and global atrophy; (2) serious cognitive impairment, memory and other cognitive dysfunctions, selecting Chinese version of the Mini‐Mental State Examination (MMSE) values to exclude mild cognitive impairment cases; (3) presence of a cardiac pacemaker; (4) unstable vital signs. Healthy controls were also recruited.

### 2.2 General data of the subjects

Age, gender, course of disease, body weight, and height were collected at enrollment for each patient, and body mass index (BMI) was calculated. Age, gender, and BMI of the healthy controls were collected.

### 2.3 Scale assessments

The patients underwent eight assessments as follows: (1) Burke‐Fahn‐Marsden Dystonia Rating Scale (BFMDRS) for WD patients with dystonia; (2) Zung's Self‐Rating Anxiety Scale (SAS) for anxiety disorder; (3) Zung's Self‐Rating Depression Scale (SDS) for depressive symptoms; (4) Bucco‐Facial‐Apraxia Assessment for apraxia of oral facial muscles; (5) Behavioral Assessment of the Dysexecutive Syndrome (BADS) for executive dysfunction; (6) Standardized Swallowing Assessment (SSA) for dysphagia; (7) video fluoroscopic swallowing study (VFSS) for dysphagia and abnormalities during the swallowing process; and (8) Frenchay Dysarthria Assessment scale (FDA) for oral motor analysis.

Measures were assessed by three trained clinicians (SR.Z., 2 years of experience; DP.C., 5 years of experience; AQ.L., > 10 years of experience) and verified by an associate chief physician (MF.H., > 30 years of experience). The average value of each assessment was taken as the final score.

### 2.4 EEG recordings

The recordings were performed in a quiet room. All participants took regular breaks, followed a reasonable diet, avoided drinking coffee or tea, and abstained from smoking or consuming alcohol.

EEG data were recorded from 19 surface Ag‐AgCl electrodes (10‐20 system) by using an EEG amplifier (Natus Medical Incorporated, USA). The electrode‐to‐skin impedances were kept below 5 kΩ (Čukić et al., 2020). The active recording electrode was placed at the central (Cz) recording site, and the reference electrode was placed in the midline of the frontal pole (Fpz). For offline corrections of electrooculogram activity, continuous EEG data were recorded during an eye calibration task. All participants were instructed to complete five different tasks: (1) relaxed and stayed awake with their eyes closed; (2) read the English alphabet; (3) swallowed liquids (2, 5, 15 ml in a single swallow); (4) swallowed 15 ml of pastes; and (5) swallowed approximately 3 g of bread after chewing. For the exact duration of each swallowing task requiring no more than 10 s, the corresponding EEG signal was recorded for 1–10 s during each processing task to ensure stationarity and comparability (Kreuzer et al., 2014; Palva & Palva, 2018).

### 2.5 EEG data processing and EEG PSE analysis

EEG data were further processed offline with automated routines using the EEGLAB12 toolkit in MATLAB 2013b software. The data were band‐pass filtered between 1 and 40 Hz and notch filtered between 49 and 51 Hz. A1 and A2 were averaged to create a reference for all channels. An independent component analysis algorithm in EEGLAB12 was employed to detect and remove the records caused by eyeblinks, horizontal eye movements, and other artifacts (Chriskos et al., 2021).

The estimate of the power spectral density (PSD) was calculated by Welch's periodogram in the toolkit. Then, total power within relevant frequency bands were obtained to provide the normalized PSD: *p*(*f*). We chose the frequency within 1 to 40 Hz of PSD and computed the corresponding normalization $\mathrm{PSD}:\hat{p}\left(f\right)=p\left(f\right)/\sum_{f=1}^{f=40}p\left(f\right)$ then estimated the power spectral entropy (PSE) based on the Shannon entropy formula: $\mathrm{PSE}=-\sum_{f=1}^{f=40}\hat{p}\left(f\right)\log\;\left[\hat{p}\left(f\right)\right]$ (Tian et al., 2017; Zhang et al., 2015).

### 2.6 Statistical methods

All data were checked for completeness and were analyzed using Statistical Package for the Social Sciences (SPSS version 26.0). Quantitative statistics for continuous variables with a normal distribution were calculated by standard tests (*p* < .05) and expressed as the mean ± standard deviation (*M* ± SD). Quantitative data with a nonnormal distribution were analyzed by using Kruskal–Wallis H tests or Mann–Whitney *U* tests and expressed as the median and lower and upper quartiles (P50 [P25, P75]). Qualitative statistics are expressed as frequencies and percentages (*n*, %), and comparisons between groups were performed using the chi‐square test. Correlations involving two normally distributed variables were analyzed by Pearson's correlation analysis, and correlations between two nonnormally distributed variables were analyzed by Spearman's rank order correlation analysis. Two‐sided *p* values of less than .05 were considered statistically significant.

## 3 RESULTS

### 3.1 Participants

Between October 2019 and January 2021, 63 participants were recruited, of whom 2 patients with elevated blood ammonia level (80∼109 μmol/L), 1 patient had a seizure within 4 weeks, 1 subject was found alcohol consumption, and 1 patient with incomplete scale assessments were excluded. Finally, a total of 58 patients were enrolled, including 34 WD patients with dystonia recruited in the case group and an age‐matched group of 24 WD patients without dystonia recruited in the control group. 20 healthy controls were enrolled (Table 1). There were no statistically significant differences in age or gender ratio among the individuals (*p* > .05). No significant differences in the course of disease between the case group and the control group (*p* > .05).

**TABLE 1:** Characteristics of participants (M ± SD)

Group (**n**)	Case group (34)	Control group (24)	Healthy group (20)	*X* ^2^/*t*/*F* value	*p* Value
Gender (male/female)	22/12	12/12	10/10	1.687^a^	.430
Age (years)	26.1 ± 5.8	27.4 ± 10.1	26.4 ± 5.1	0.225^b^	.799
Disease course (years)	7.66 ± 5.01	8.48 ± 5.46	/	−0.592^c^	.556

^a^ *X* ^2^ value.

^b^ *F* value.

^c^ *t* value.

### 3.2 BMI analysis

The mean BMI in the case group (18.75 ± 3.22 kg/m^2^) was significantly lower than that in the control group (20.84 ± 3.08 kg/m^2^) and in the healthy group (22.08 ± 2.49 kg/m^2^) (*p* < .05). There was no significant difference in BMI between the control group and the healthy group (*p* > .05) (Table 2).

**TABLE 2:** Comparison of BMI in participants (M ± SD)

Group (n)		BMI	*p* Value
Case group (34)	Control group (24)	18.75 ± 3.22	<.05
	Healthy group (20)	20.84 ± 3.08	<.05
Control group (24)	Healthy group (20)	22.08 ± 2.49	.157

### 3.3 Emotional disorders and orofacial apraxia in all WD patients

The higher the scores of SAS, SDS and Bucco‐Facial‐Apraxia Assessment prompt the worse the symptoms of the patients. The case group showed significantly higher SAS, SDS, and Bucco‐Facial‐Apraxia Assessment scores than those in the control groups (*p* < .05) (Table 3).

**TABLE 3:** Comparison of SAS, SDS, Bucco‐Facial‐Apraxia Assessment, and BADS scores in the case group and control group (M ± SD/P50 [P25, P75])

Variables	Case group (34)	Control group (24)	*p* Value
SAS	49.93 ± 7.83	43.32 ± 8.61	<.05
SDS	55 (51.25, 61.56)	44.38 (35.50, 48.44)	<.05
Bucco‐Facial‐Apraxia Assessment	2 (1, 3.25)	0 (0, 0)	<.05
BADS	9.71 ± 3.15	14.88 ± 3.67	<.05

In the case group, 15 patients (44.12%, 15/34) scored above the cutoff point for the presentation of anxiety and 21 of them suffered from depressive syndrome (61.76%, 21/34). In contrast, 5 were considered as with anxiety (20.83%, 5/24), and 4 patients were estimated as depressed (16.67%, 4/24) in the control group.

### 3.4 Executive functions in all WD patients

**FIGURE 1:**
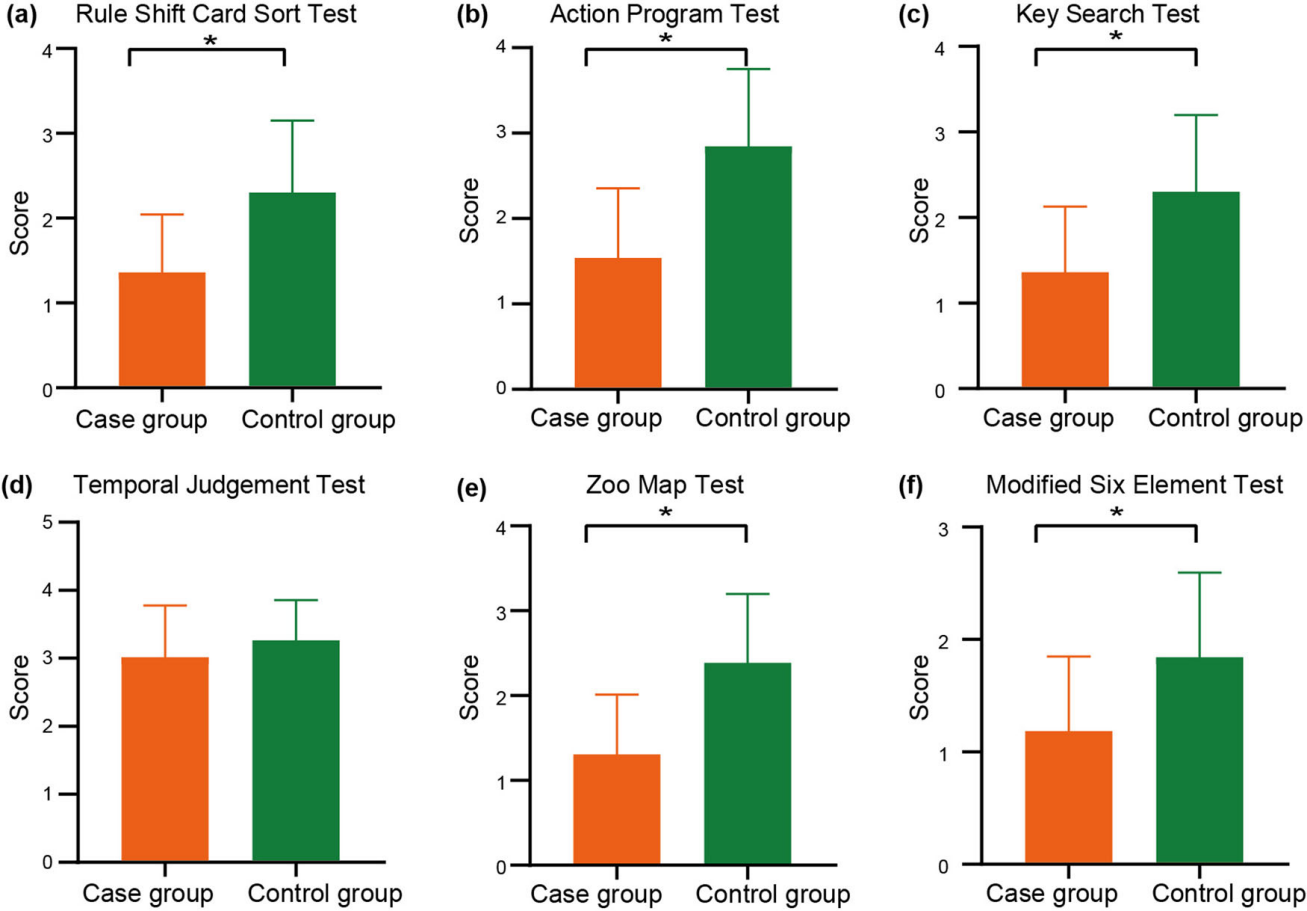
Comparison of BADS subscales between the case group and the control group (*p < .05). (a) The rule shift card sort test, (b) the action program test, (c) the key search test, (d) the temporal judgment test, (e) the zoo map test, and (f) the modified six elements test

The low scores of the BADS and its subscales suggest the more sever executive dysfunction. The total BADS scores and the subscales (the rule shift card sort test, the action program test, the key search test, the zoo map test, and the modified six elements test) in the case group were lower than those in the control group (*p* < .05; Table 3 and Figure 1). There was no difference in the temporal judgment test (*p* = .22).

### 3.5 VFSS assessment in the case group

**FIGURE 2:**
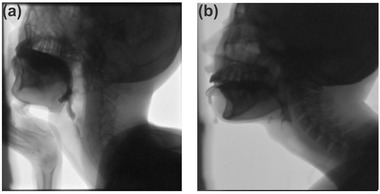
VFSS captures before swallowing and during swallowing. (a) Before swallowing. The liquid flowed from the mouth and could not be held in the anterior portion of the oral cavity but flowed to the oropharynx during the preparatory stage. (b) During swallowing. During the oral and pharyngeal stages, the tongue could not propel liquid posteriorly into the oropharynx, but the liquid flowed following chin lifting. Aspiration was primarily associated with abnormal epiglottis

In the case group, almost all of the patients presented with dysarthria (94.11%, 32/34), and 24 out of 34 (70.59%) patients suffered from dysphagia. The proportions of dysarthria and dysphagia in WD patients were 55.17% (32/58) and 41.38% (24/58), respectively. Eight of the case groups were examined with VFSS. Deglutition abnormalities were found mainly relating to the oral preparatory stage and oral stage, during which the lips, tongue, and upper and lower jaws moved incongruently (Figure 2a). In addition, the soft palate was elevated, and the epiglottis deflected abnormally during the pharyngeal stage (Figure 2b). The esophageal stage of swallowing was regular.

### 3.6 Correlation analysis of dystonia

**FIGURE 3:**
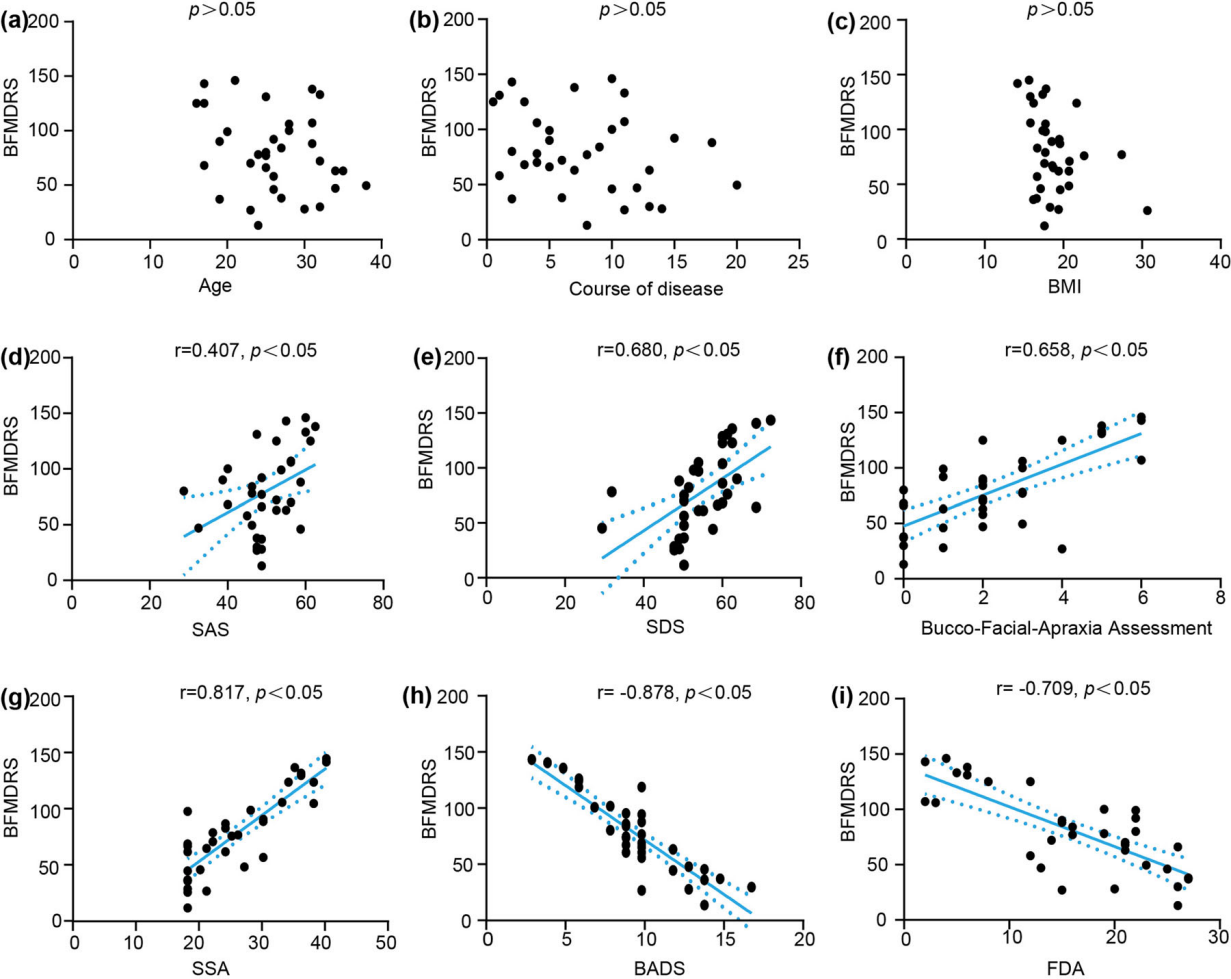
Correlations between BFMDRS scores and (a) age, (b) course of disease, (c) BMI, (d) SAS scores (*r* = .407, *p* < .05, 95% confidence interval (CI) 0.080 to 0.655), (e) SDS scores (*r* = .680, *p* <.05, 95% CI 0.435 to 0.831), (f) Bucco‐Facial‐Apraxia Assessment scores (*r* = .658, *p* < .05, 95% CI 0.435 to 0.831), (g) SSA scores (*r* = .817, *p*<.05, 95% CI 0.657 to 0.907), (h) BADS scores (*r* = −.878, *p* < .05, 95% CI −0.938 to −0.767), and (i) FDA scores (*r* = −.709, *p* < .05, 95% CI −0.847 to −0.479) in the case group

Increasing scores of BFMRDS, SAS, SDS, SSA, and Bucco‐Facial‐Apraxia Assessment indicate worsening of symptoms. While the decreasing scores of BADS and FDA reflect the worsening symptoms. In this study, BFMRDS scores correlated positively with SAS scores (*r* = .407, *p* < .05; Figure 3d), SDS (*r* = .608, *p* < .05; Figure 3e), Bucco‐Facial‐Apraxia Assessment scores (*r* = .658, *p* < .05; Figure 3f) and SSA (*r* = .817, *p* < .05; Figure 3g). And the BFMRDS scores of WD patient with dystonia were negatively correlated with BADS scores (*r* = −.878, *p* < .05; Figure 3h) and FDA scores (*r* = −.709, *p* < .05; Figure 3i). There were no significant correlations between dystonia and age, BMI or disease course (Figure 3a–**c**).

### 3.7 EEG PSE for all participants in different tasks and stages

**FIGURE 4:**
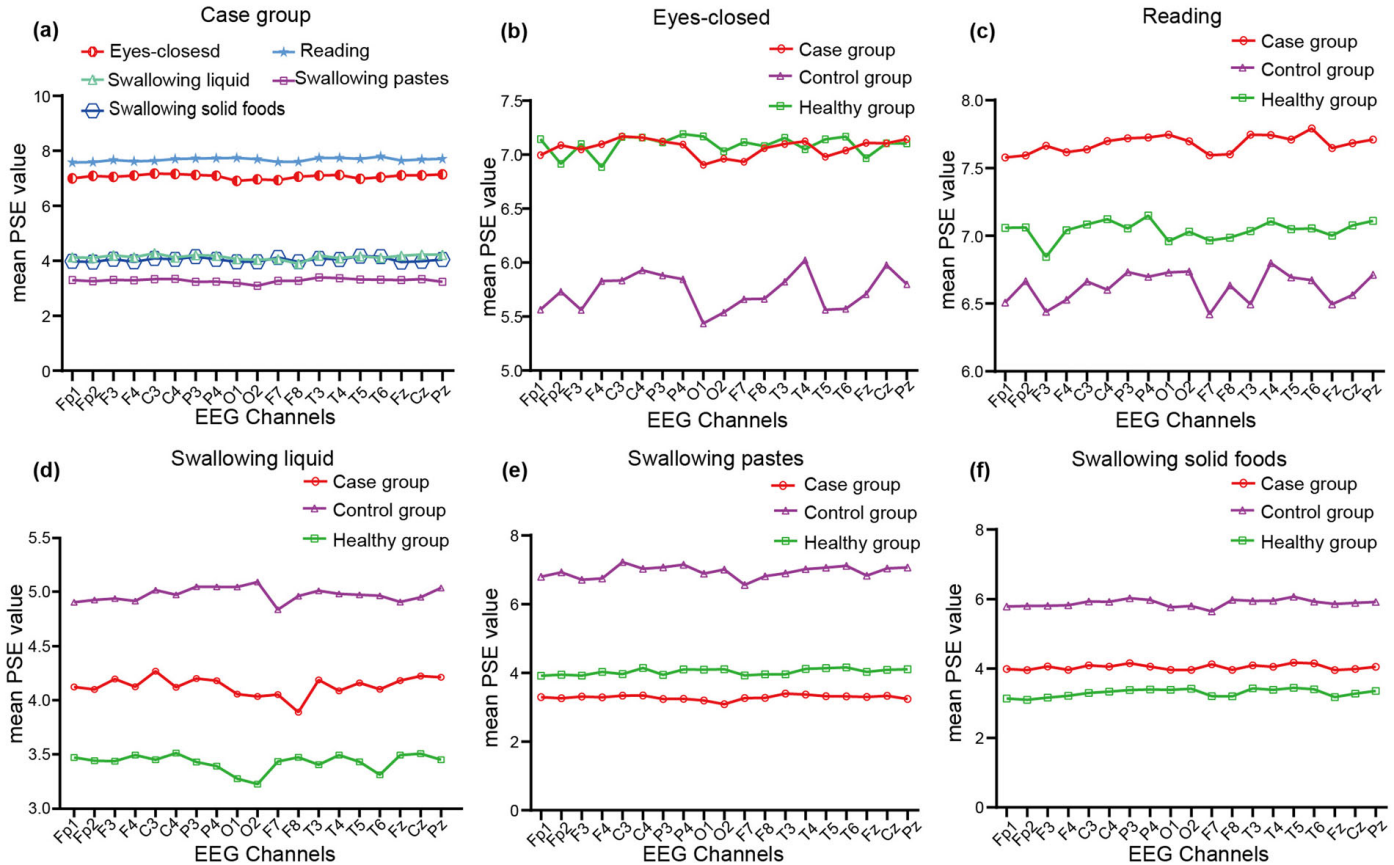
The overall PSE value of the case group and comparisons of mean PSE values across the 5 tasks in 3 groups for each EEG channel. (a) The mean PSE values in 5 tasks of the case group. (b) The mean PSE values in eyes‐closed task. (c) The mean PSE values in reading state. (d) The mean PSE values in swallowing liquid. (e) The mean PSE values in swallowing paste. (f) The mean PSE values in swallowing solid foods

Higher PSE value represents the more complex the neural dynamics. Figure 4a shown the mean PSE values of five tasks in the case group. In the eyes‐closed task, the mean PSE values of the case group were higher than those in the control group (Figure 4b). The PSE values of the case group were the highest in the reading state (Figure 4c) but were the lowest in the swallowing paste stage than those in the controls (Figure 4e). Furthermore, in the swallowing liquid and solid food stages, the PSE values of the case group ranked middle among the three groups (Figure 4d and f).

## 4 DISCUSSION

ATP7B, encoding by *ATP7B*, is a copper‐transporting P‐type ATPase (Shribman et al., 2021). WD is related to *ATP7B* mutations, which causing excessive copper accumulation in tissues and organs (Li et al., 2020; Yuan et al., 2021). As the pathophysiological basis of dystonia in WD, basal ganglia are more sensitive to copper toxicity (Yuan et al., 2021).

The WD patients with dystonia had a significantly decreased BMI (Table 2). We assumed the reasons for the lower BMI in the case group may be caused by the following factors: (1) dystonia increased energy expenditure due to involuntary muscle contractions (Williams et al., 2013); (2) patients needed assistance with eating because of their postural inflexibility, and those with dysphagia had greater difficulty in food intake leading to malnutrition (Rodd et al., 2021); and (3) the striatal dopaminergic system controlled by hypothalamus modulating food intake was impacted in WD patients (Folgueira et al., 2019; Kistner et al., 2014).The exact mechanisms for low BMI of WD patients with dystonia warrant further study.

The symptoms of anxiety and depression in the case group were more severe than the control group (Table 3). We suspected that dystonia had a great influence on quality of life. Otherwise, WD patients with dystonia may have dysmorphic features, and they tended to suffer from shame‐related social and psychological disorders. In addition, dystonia is a circuit disorder involving the cortex which plays a crucial role in emotion (Anastasiades & Carter, 2021; Hare & Duman, 2020; Mencacci et al., 2020). The pathophysiologic association of dystonia with mood disorders might explain the comorbidity of anxiety and depression in WD patients with dystonia (Mencacci et al., 2020).

According to the Bucco‐Facial‐Apraxia assessment, we found most of WD patients with dystonia were unable to perform some functional movements such as pursing or puckering of the lips. Lateral precentral gyrus and prefrontal cortex are associating with orofacial motor action (Hashimoto et al., 2021; Loh et al., 2020). Damage of the premotor cortex, parietal association cortex or supplementary motor cortex may produce higher‐order deficits and apraxia (Attallah et al., 2020). Dystonia is triggered or worsened by action initiation facilitated by the premotor cortex (Ribot et al., 2019). Thereby, we assumed that copper accumulation and neuropathologic abnormalities of frontal cortical and subcortical structures contributes to orofacial apraxia in WD patients with dystonia.

The BADS was conducted to assess executive functions in the WD patients. The subscales of the BADS represented different domains (Murakami et al., 2017): the rule shift card sort test measures cognitive flexibility; the action program test assesses action planning skills; the key search test identifies deterioration regarding planning and monitoring progress; the temporal judgment test estimates temporal memory; the zoo map test assesses planning; and the modified six elements test examines especially planning function. The total BADS scores and scores of five subscales without the temporal judgment test were significantly lower in the case group than those in the control group (Table 3 and Figure 1a–c, e and f). The lower the scores represented the more severe executive dysfunction. The patients with dystonia may had difficulty finishing the tasks because of their inflexible movements. The more important reason was that the impairments in the prefrontal cortex (Leśniak et al., 2022). Highly sensitive to the dopaminergic environment, the prefrontal cortex is also related to executive functions (Ott & Nieder, 2019; Uddin, 2021). Research has shown that working memory impairments are severe in monkeys with dopamine depletion in the prefrontal cortex and are as severe as those in monkeys with complete ablations of the prefrontal cortex (Brozoski et al., 1979; Cools et al., 2019; Froudist‐Walsh et al., 2021). In addition, striatal dopamine and dopamine D2Rs in the striatum has been found reduced in WD patients (Roy et al., 2018). Dopamine not only modulates cognitive control through the prefrontal cortex but also alters incentive motivation through the striatum, which then affects cognitive functioning (Cools et al., 2019; Westbrook & Frank, 2018). Furthermore, basal ganglia are more vulnerable to copper toxicity in WD. Therefore, it is reasonable to infer that executive dysfunction in WD patients with dystonia may be exerted by the metal accumulation (Li et al., 2020) and alterations in the prefrontal cortex, basal ganglia and its neurotransmitter dopamine.

The proportions of dysarthria and dysphagia in the WD patients were 55.17% (32/58) and 41.38% (24/58), respectively, which were consistent with the previous literature (Litwin et al., 2017). But the patients with dystonia had higher proportion of dysarthria (94.11%) and dysphagia (70.59%). Eight patients who underwent VFSS showed abnormal findings in the oral preparatory stage, oral stage, and pharyngeal phase. Based on the VFSS, the patients with dysphagia mainly manifested abnormalities in the oral preparation and oral phases (Figure 2). It has been suggested that the primary and secondary sensorimotor cortices are involved in the oral preparatory and oral stages when swallowing occurs (Ertekin, 2011; Shaw & Martino, 2013). By means of dopaminergic mechanisms, the amygdala and lateral hypothalamus are thought to promote swallowing (Alfonsi et al., 2021; Muhle et al., 2021). Thus, we assumed that abnormal activity in the basal ganglia, dopaminergic system, primary, and secondary sensorimotor cortices may be associated with dysarthria. Moreover, it has been claimed that the activation of the cortical component related to orofacial movements overlaps with the activation of the cortical component related to swallowing (Jestrović et al., 2015). Therefore, orofacial apraxia and abnormal executive function of the vocal system may also contribute to dysarthria and dysphagia in the WD patients with dystonia.

Correlation analyses revealed that anxiety, depression, apraxia, executive dysfunction, dysarthria and dysphagia were associated with dystonia (Figure 3). The more severe the dystonia often accompanied with more obvious symptoms. These correlations, in turn, indicated that the basal ganglia‐cerebello‐thalamo‐cortical circuit is an integrated network that operates over the domains of movement, emotion, and cognition (Bostan & Strick, 2018). In this network, even small changes in a single node can permeate the entire network and thus promote dysfunction. Copper dramatically accumulate in the circuit, especially in cortex and basal ganglia (Shribman et al., 2021). Copper accumulation can lead to not only cardiolipin fragmentation of the mitochondrial membrane causing mitochondrial dysfunction but ceruloplasmin reduction causing iron metabolism disorder(Li et al., 2020; Shribman et al., 2021; Yuan et al., 2021). These may contribute to the neuropathological changes including demyelination, neuronal loss, spongiosis, cavitation and reactive astrogliosis in WD and finally manifest a wide range of symptoms(Shribman et al., 2021; Yuan et al., 2021). Furthermore, as high‐level neurological functions, executive functions may play important roles in the pathogenesis of dystonia, dysphagia and dysarthria.

The brain signal complexity can reflect the state of neural networks. Chaotic dynamics has been considered a mechanism for balancing cortical variability (Muscinelli et al., 2019). Chaos is a normal phenomenon in the context of human consciousness activities. A significant increase in the complexity of brain signals, namely, a higher degree of chaotic activity, is related to states of consciousness (Boncompte et al., 2021; Wang et al., 2010). This may indicate that the brain needs to handle varied sensorimotor information (Keshmiri, 2020; Mateos et al., 2018). The complexity of cortical activity is positively correlated with entropy (Keshmiri, 2020). PSE analyze the variations in the characteristics of brain activity signals. The higher the PSE value means more complex the neural dynamics.

The Figure 4a shows the overall PSE value in five stages of WD dystonia. We found that the PSE values in the case group were higher than those in the control group during the eye‐closed task (Figure 4b) and were the highest in the reading task (Figure 4c) among three groups. It has been recognized that the excitability of the brain will increase during wakefulness and reading for the increase in visual sensory input and vocal motor output (Wang et al., 2010). To keep eyes closed or to finish reading, a large amount of internal (e.g., the desire to complete the task) and external (e.g., sensory stimuli) information was delivered to the brains for integration and segregation. Meanwhile, due to the loss of inhibition at neuronal circuits in dystonia (Balint et al., 2018), the cerebral electrical activity may be scattered, irregular, and arbitrary. These may contribute to complex cortex activity, thus, PSE values increased.

In the swallowing paste stage, the PSE values of the case group were the lowest among 3 groups (Figure 4e), while in the swallowing liquid and solid foods stages, they ranked intermediate (Figure 4d and f). When one is concentrated to perform an action task, the brain activity is high synchrony (Mateos et al., 2018). Therefore, fewer permutation vectors are required to quantize the signals, and the PSE values decreased (Keshmiri, 2020; Mateos et al., 2018). In addition, during swallowing, cortical initiation is involved only in triggering deglutition and the beginning of the oral stage, and no further cortical activation is needed in the subsequent stages (Ertekin, 2011; Jestrović et al., 2014). The demand for cerebral synaptic activity may decrease after the swallowing trigger; hence, the PSE values may be decrease. Importantly, the network disorder cannot be ignored, which pathologically decreases swallowing signals. And it cannot be ruled out that fewer neurons are available for managing and transmitting information led by neural network dysfunction (Mateos et al., 2018). This variability reflected lower complexity and lower PSE. Additionally, pastes adhere to the oral mucosa and flow slower than liquid, thus, the bolus transit time and overall airway protection increase (Jestrović et al., 2015, 2013). These factors make pastes easier to gulp. The patients may feel more relaxed to finish swallowing. Thus, the PSE values further decreased.

To the best of our knowledge, this is the first prospective study to explore the correlation between dystonia and several neurobehavioral disabilities in WD patients. And it is the first time to apply the PSE of EEG to detect irregularities and the complexities of cortical synaptic activity in WD patients with dystonia. Apart from de‐coppering therapy for WD patients with dystonia, the treatment to their nutrition, emotional state and muscle tone is equally important. Inspired by the impairment of the high‐level cognitive process, we consider the therapy targeting executive function is necessary in WD with dystonia.

Nevertheless, the limitations of this study should be acknowledged. First, the sample size was relatively small. Second, the EEG were vulnerable to disturbance, thus, some important electroencephalographic information may be magnified or not available. Further studies with larger sample sizes are needed to validate our results.

## 5 CONCLUSION

Accompanied with lower BMI, WD patients with dystonia were more vulnerable to anxiety, depression, apraxia, dysarthria, dysphagia, and executive dysfunctions. Their cortical electrical activity remarked a notable chaos while eyes‐closing and reading with a concomitant reduction during the swallowing. These alterations were related to high‐level neurological functions, which worth further investigation. Meanwhile, it is hoped that this analysis may help providing a new insight for WD dystonia.

## AUTHOR CONTRIBUTIONS

AL and MH conceived and designed the study; AL won the funding; SZ, QZ, and DC contributed to study design, data collection, data analysis and interpretation, and drafting the manuscript; SZ, JC, and ZH contributed to electroencephalography collecting and analysis; ZZ and QY were responsible for research supervision and manuscript revision; MH and ZP provided suggestions for revision and proof of the manuscript. All authors approved the final version for submission.

## CONFLICT OF INTEREST

The authors declare that they have no conflict of interest.

### PEER REVIEW

The peer review history for this article is available at: https://publons.com/publon/10.1002/brb3.2791.

## Data Availability

Data are available upon reasonable request.
